# A Standardized Analysis of Tertiary Lymphoid Structures in Human Melanoma: Disease Progression- and Tumor Site-Associated Changes With Germinal Center Alteration

**DOI:** 10.3389/fimmu.2021.675146

**Published:** 2021-06-24

**Authors:** Franziska Werner, Christine Wagner, Martin Simon, Katharina Glatz, Kirsten D. Mertz, Heinz Läubli, Johannes Griss, Stephan N. Wagner

**Affiliations:** ^1^ Laboratory of Molecular Dermato-Oncology and Tumor Immunology, Department of Dermatology, Medical University of Vienna, Vienna, Austria; ^2^ Institute of Medical Genetics and Pathology, University Hospital Basel, University Basel, Basel, Switzerland; ^3^ Institute of Pathology, Cantonal Hospital Baselland, Liestal, Switzerland; ^4^ Laboratory for Cancer Immunotherapy, Department of Biomedicine and Medical Oncology, Department of Internal Medicine, University Hospital Basel, Basel, Switzerland; ^5^ Department of Dermatology, Medical University of Vienna, Vienna, Austria

**Keywords:** Tertiary Lymphoid Structures (TLS), TLS maturation, tumor microenvironment, multiplex immunohistochemistry (mIHC), germinal center polarity, spatial distribution, automated imaging and analysis, human melanoma

## Abstract

There is increasing evidence that tertiary lymphoid structures (TLS) control not only local adaptive B cell responses at melanoma tumor sites but also the cellular composition and function of other immune cells. In human melanoma, however, a comprehensive analysis of TLS phenotypes, density and spatial distribution at different disease stages is lacking. Here we used 7-color multiplex immunostaining of whole tissue sections from 103 human melanoma samples to characterize TLS phenotypes along the expression of established TLS-defining molecular and cellular components. TLS density and spatial distribution were determined by referring TLS counts to the tissue area within defined intra- and extratumoral perimeters around the invasive tumor front. We show that only a subgroup of primary human melanomas contains TLS. These TLS rarely formed germinal centers and mostly located intratumorally within 1 mm distance to the invasive tumor front. In contrast, melanoma metastases had a significantly increased density of secondary follicular TLS. They appeared preferentially in stromal areas within an extratumoral 1 mm distance to the invasive tumor front and their density varied over time and site of metastasis. Interestingly, secondary follicular TLS in melanoma often lacked BCL6^+^ lymphatic cells and canonical germinal center polarity with the formation of dark and light zone areas. Our work provides an integrated qualitative, quantitative and spatial analysis of TLS in human melanoma and shows disease progression- and site-associated changes in TLS phenotypes, density and spatial distribution. The frequent absence of canonical germinal center polarity in melanoma TLS highlights the induction of TLS maturation as a potential additive to future immunotherapy studies. Given the variable evaluation strategies used in previous TLS studies of human tumors, an important asset of this study is the standardized quantitative evaluation approach that provides a high degree of reproducibility.

## Introduction

Immune checkpoint blockade (ICB) aims to overcome inhibition of anti-tumor T cell effector functions in an inflamed but immunosuppressed tumor microenvironment (TME, *i.e.* tumor cells with surrounding extracellular matrix and stromal/immune host cells). That way, ICB has transformed the therapy of many cancer types, particularly of melanoma, where ICB has led to progression-free survival rates of 36% at 5 years in metastatic patients (1). Yet, the other 64% of patients experience disease progression and alternative therapeutic options are naught.

The outcome of ICB therapy in cancer patients has been linked to immune cell infiltration into the TME and the quality and magnitude of the induced activation of immune cells including T cells, NK cells and, more recently tumor-associated B cells. In human melanoma, up to 33% of the immune cells found in the TME can be B cells. Though B cells in human cancer have been shown to express immunoinhibitory cytokines, growth factors and cell surface molecules that impede anti-tumor immune and drug responses (2, 3), several reports suggest a role in supporting tumor immunity and limiting disease progression. They demonstrate a positive prognostic association of CD20^+^ B cell numbers in primary human melanoma (4, 5) and, together with increased CD138^+^ plasma cell numbers, in metastatic human melanoma (6). In line with these data, B cells have recently been shown to sustain inflammation and CD8^+^ and CD4^+^ T cell numbers in the TME of human melanoma, and to directly augment T cell activation by ICB (7). Furthermore, B cell markers were reported to be increased in tumor samples from responders to neoadjuvant ICB compared with non-responders in patients with high-risk resectable melanoma (8), and pretreatment B cell counts, particularly of plasmablast-like B cells, have been found to predict response and survival in metastatic melanoma patients receiving ICB (7).

Further independent comparative analyses of human melanoma and soft tissue sarcoma described higher numbers of mature tertiary lymphoid structures (TLS) in tumor samples from patients who responded to ICB treatment compared to non-responding patients (9–11). As ectopic lymphoid structures at tumor sites, TLS share many structural and functional features with canonical lymphoid structures from secondary lymphoid organs which are important drivers of adaptive B cell and T cell responses. Consistently, higher numbers of mature TLS are associated with clonal B cell expansion, increased B cell receptor diversity and increased frequency of class-switched memory, plasmablast/plasma cell-like and activated CD69^+^ B cells in human melanoma (9, 10). Again, the number of intratumoral B cells before treatment, either together with increased or independent of CD8^+^ T cell numbers, was shown to be predictive for improved patient survival (9). Together with the reported clonal amplification, somatic hypermutation and isotype switching of B cells in microdissected lymphoid follicles from human melanoma skin metastases (12) and our observation on the loss even of CD20^-^ B cells from the human melanoma TME upon depletion of TLS by anti-CD20 therapy (7), these data strongly support the concept of TLS as the main generator of local adaptive B cell responses in melanoma. In addition, there is increasing evidence that TLS can also play a significant role in the induction of local adaptive T cell responses in human melanoma. Mature TLS also contain T cell zones where mature dendritic cells and most likely mature B cells present antigenic peptides to CD4^+^ and CD8^+^ T cells (13–16). Consistent with this, the density of mature dendritic cells is associated with strong infiltration by activated T cells and favorable survival in primary human melanoma (17) and CD3^+^ T cells from inside TLS of human melanoma metastases show a higher expression of T cell activation markers compared with T cells from outside (10). Moreover, in TLS-enriched melanoma metastases, increased immune signatures for antigen presentation and processing, T cell receptor signaling and differentiation of T helper 1 and 2 cells were found (10), whereas in TLS-depleted melanomas, T cell signatures indicated a more dysfunctional state (9).

Similar to canonical lymphoid structures in secondary lymphoid organs, phenotypic changes of TLS have been described in human cancer and are associated with a variable composition of diverse cell types with distinct differentiation. Here, the cellular composition of TLS appears to vary between patients and cancer types, stages and sites (18–22). While there are some reports on the presence of distinct TLS-associated cell types in primary human melanoma (17, 23) and a smaller cohort of human melanoma skin metastases (12), a systematic comparative analysis of TLS phenotypes, their cellular composition and spatial distribution in human melanoma samples from different disease stages and sites is still lacking. Given the high variability of evaluation approaches used by previous studies for TLS definition and quantification, we applied 7-color multiplex immunohistochemistry to characterize TLS phenotypes along the coordinated expression of established TLS-defining molecular and cellular components together with a standardized evaluation strategy specifically adapted for high reproducibility. We found progression-, tumor site-, but not prognosis-associated changes in TLS phenotypes, density and spatial distribution. In contrast to secondary lymphoid organs, secondary follicular melanoma TLS showed a remarkable paucity of canonical germinal center formation.

## Materials and Methods

### Patient Cohorts

#### Primary Human Melanomas and Matched Early/Regional Metastases

Whole tissue sections were obtained from routine formalin-fixed paraffin-embedded (FFPE) blocks of cutaneous primary melanomas from Caucasian patients who underwent surgery between the years 2002 and 2014 at the Cantonal Hospital Baselland, Liestal (5). All tumor samples were obtained with informed patients’ consent and the pathology files retrieved as approved by the local Ethics Committee (EKNZ vote BASEC 2016-01499). Histological diagnoses were made by board-certified pathologists from the Cantonal Hospital under the guidance of KM. This cohort included 48 patients with primary cutaneous melanoma. 27 patients presented without metastasis within a follow-up interval of up to 140 months (mean: 42 months, **Table 1**); 21 patients were diagnosed with regional metastasis at the time of first diagnosis (**Table 2**).

**Table 1:** Clinical and histopathological summary of melanoma patients without metastasis.

Number of patients		27
**Follow-up (months**)	Mean	42
	Median	28
	Range	8-140
**Age (years)**	Mean	67
	Median	70
	Range	31-93
**Breslow depth (thickness in mm)**	Mean	2,73
	Median	1,99
	Range	0,36-10
**Location**	Extremities	8
	Head/Neck	2
	Trunk	17
**Ulceration**	Present	10
	Absent	17
Histotype*	SSM	20
	NM	6
	NOS	1
**Sex**	Female	11
	Male	16

*SSM, superficial spreading melanoma; NM, nodular Melanoma; NOS, not otherwise specified.

**Table 2:** Clinical and histopathological summary of melanoma patients with metastasis at the time of first diagnosis.

Number of patients		21
**Age (years)**	Mean	66
	Median	67
	Range	31-91
Breslow depth (thickness in mm)*	Mean	6,30
	Median	5,73
	Range	1-15
Location*	Extremities	7
	Head/Neck	3
	Trunk	10
Ulceration*	Present	15
	Absent	5
Histotype**	SSM	6
	NM	15
**Sex**	Female	6
	Male	15

*Three samples without information about Breslow depth, one about location, one about ulceration.

**SSM, superficial spreading melanoma; NM, nodular melanoma.

From 10 of the latter patients additional 16 metastatic samples could also be included in our analysis. These early metastatic samples almost exclusively consisted of locoregional skin (n=4) and clinically detectable (macroscopic) nodal metastases (n=11, **Table 3**) where tumor deposits had completely or almost completely replaced lymph node tissue. None of these patients had received local or systemic antitumor treatment before surgery.

**Table 3:** Numbers of biopsy sites of melanoma patients with early and late metastases.

Biopsy site	Number of patients	
	early metastases	late metastases
Sum	16	39
Skin	4	15
Lymph node	11	13
Lung	1	4
Brain	0	3
Nerve	0	1
Kidney	0	1
Bone	0	1
Bladder	0	1

#### Late/Distant Metastases

In addition, we analyzed whole sections from 39 distant human melanoma metastases, mainly derived from cutaneous (n=15) and lymph node (n=13) sites (**Table 3**). These samples were collected between the years 2015 and 2019 at the University Hospital Basel. Tumor samples were obtained with informed patients’ consent and the pathology files retrieved as approved by the local Ethics Committee (EKNZ vote BASEC 2019-00927). Histological diagnoses were made by board-certified pathologists from the Institute of Pathology, University Hospital Basel, under the guidance of KG. In lymph node metastases, tumor deposits had completely or almost completely replaced lymph node tissue. Desmoplastic subtypes of melanoma were not included in this study as they show a distinct clinical behavior (24).

### Seven Color Multiplex Immunohistochemical Staining

Tumor tissue analysis and read-out were approved by the Ethics Committee of the Medical University of Vienna (ethics vote 1999/2019). First, each of the following antibodies was established on four-micrometer sections from FFPE tissue of human tonsil: CD20 (mouse monoclonal IgG2a, clone L26, 1:2000, Agilent, M0755), CD4 (mouse monoclonal IgG1, clone 4B12, 1:500, Agilent, M7310), CXCL13 (rabbit polyclonal IgG, 1:1000, Proteintech, 10927-1-AP), CD21 (rabbit polyclonal IgG, 1:3600, Proteintech, 24374-1-AP), CD23 (rabbit monoclonal IgG, clone SP23, 1:900, Novus Biologicals, NB120-16702) and BCL6 (mouse monoclonal IgG1, clone 1E6B1, 1:24000, Proteintech, 66340-1-Ig). Each antibody was assigned to one of the fluorophores Opal 520, Opal 540, Opal 570, Opal 620, Opal 650 and Opal 690 (Akoya Biosciences) diluted in 1X Plus Amplification Diluent (Akoya).

For multiplex stainings, these antibodies were successively applied to deparaffinized sections from FFPE tumor samples in six iterative rounds of immunostaining. Each round started with heated antigen retrieval with either citrate buffer (pH 6.0) or Tris-EDTA (pH 9.0) for 30 min, fixation with 7.5% neutralized formaldehyde (SAV Liquid Production) and a 15 min blocking step using 20% normal goat serum (Agilent, X0907). Thereafter, primary antibodies were applied followed by incubation with respective biotinylated anti-mouse/-rabbit secondary antibodies (Agilent, K5003), Streptavidin-HRP (Agilent, K5003) and Opal fluorophore dye (Akoya). After six rounds of immunostainings, nuclei were counterstained with DAPI (PerkinElmer, FP1490). A detailed protocol of the immunohistochemistry staining procedure was previously described by us (7).

Tyramid signal amplification-based visualization of the primary antibodies was established on control tonsil tissue, the gold standard for lymphocyte antigen detection in pathology, and the signal balanced by diluting the primary antibodies to obtain staining levels and cell frequencies comparable to conventional immunofluorescence staining. Negative controls included the use of isotype instead of primary antibodies and stainings without primary antibodies. Single antibody stainings were run in parallel to control for false positive (incomplete stripping of antibody-tyramide complexes) and false negative results (antigen masking by multiple antibodies, “umbrella-effect”) as well as for spillover effects (detection of fluorophores in adjacent channels), again as described by us before (7).

Additional immunofluorescent double stainings were performed with antibodies against BCL6 (mouse monoclonal IgG1, clone 1E6B1, 1:24000, Proteintech, 66340-1-Ig)-Opal 650 and Ki67 (mouse monoclonal IgG1, clone MIB-1, 1:1600, Agilent, M7240)-Opal 570.

### Automated Acquisition and Quantification of TLS

Multiplexed slides of the whole tissue sections were scanned on the Vectra 3 Automated Quantitative Pathology Imaging System (version 3.0.5., Akoya) and potential TLS were acquired in 20x magnification for downstream analysis with the tissue analysis software inForm^®^ (version 2.4.1, Akoya). This procedure included first a spectral unmixing for each specific fluorophore (7, 25, 26), followed by a trainable tissue segmentation to distinguish between different TLS phenotypes. Autofluorescence was determined on an unstained representative tumor section.

TLS phenotypes were determined based on molecular and cellular marker composition and structural features along the lines of (27, 28) with some additional specifications for secondary follicular TLS. Early TLS/dense CD20^+^ lymphocyte aggregates were trained as CD20^+^ B cell aggregates with CXCL13^+^ cells and the presence or absence of interspersed CD4^+^ T cells (CD21^-^, CD23^-^, BCL6^-^). Primary follicular TLS were trained as CD20^+^ B cell aggregates interspersed with CD4^+^ T cells and the presence of a network with CD21^+^ immature follicular dendritic cells (CD23^-^, BCL6^-^). Secondary follicular TLS were trained as structures with an additional network of CD23^+^ mature follicular dendritic cells and the presence of CD20^+^ B cell aggregates interspersed with CD4^+^ T cells without (BCL6^-^) or with the accessory expression of BCL6 in lymphatic cells (BCL6^+^). We did not use additional established markers such as DC-LAMP and PNAD, because DC-LAMP^+^ dendritic cells and PNAD^+^ high endothelial venules have been shown in different TLS phenotypes at a similar frequency as well as in lymphocyte-rich areas without apparent organization into TLS (28). Tissue segmentation quality was manually monitored for each image by the sample analyst (FW) who was blinded for sample identity at the time of image analysis.

Early, primary and secondary follicular TLS phenotypes were identified by tissue segmentation and analyzed for density (number per mm^2^ tissue area) and relative area (mm^2^ per mm^2^ tissue area) in whole tissue sections. In secondary follicular TLS, we performed an additional sub-analysis for the area of BCL6^+^ and BCL6^-^ germinal centers, respectively. Spatial distribution of TLS was manually annotated with the digital pathology image analysis software QuPath [version 0.2.3 (29)] to intra- and peritumoral perimeters of increasing radiuses (1 – max. 6 mm) drawn around the tumor-invasive front with the help of a publicly available script (30, 31) with minor adaptations to our needs. Counts and relative area of each TLS phenotype were then normalized to the tissue area (mm^2^) within the respective intra- and extratumoral perimeters. TLS overlapping two perimeters were assigned to the perimeter with the larger share. Regions of necrosis, ulceration and distinct hemorrhage were excluded from analysis as was non-infiltrated subcutaneous adipose tissue below primary melanomas due to the consistent absence of a recognizable lymphocyte infiltration.

### Statistical Analysis

All statistical analyses were performed using R (version 4.0.3). As the data was generally not normally distributed, differences between two groups were assessed using the Wilcoxon rank sum test. Differences between multiple groups were assessed using the Kruskal-Wallis test. The absolute number of samples with and without TLS between two groups was assessed using Fisher’s exact test. All p-values were corrected for multiple testing using the Benjamini & Hochberg method (abbreviated as “FDR” throughout the manuscript). Plots were created using ggplot2 [version 3.3.3 (32)], and GraphPad Prism (version 8.0.1). Statistical comparison of spatial distribution used data for the intratumoral compartment from 1 and >1 mm perimeters and focused on data for the extratumoral compartment from 1 and 2 mm perimeters, as around half of the samples presented without TLS within the 3 and higher mm extratumoral perimeters.

## Results

### Experimental Strategy

**Figure 1:**
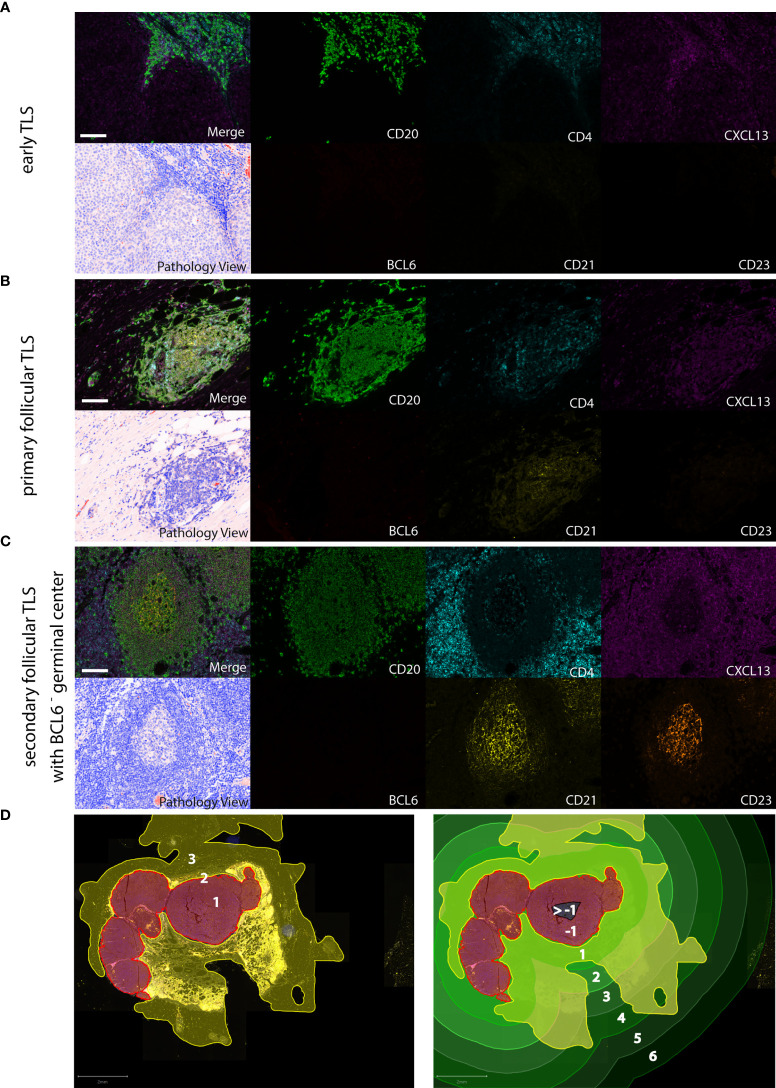
Detection of different TLS phenotypes by 7-color multiplex immunohistochemistry in human melanoma tissues. Examples for **(A)** early TLS: dense CD20^+^ lymphocyte aggregates with CXCL13^+^ cells and the presence of some interspersed CD4^+^ T cells; **(B)** primary follicular TLS: CD20^+^ lymphocyte aggregates interspersed with CD4^+^ T cells and the presence of a CD21^+^ but CD23^-^ dendritic network, surrounded by CXCL13 expressing cells; **(C)** secondary follicular TLS with a BCL6^-^ germinal center: CD21^+^ and CD23^+^ dendritic networks within CD20^+^ lymphocyte aggregates with interspersed CD4^+^ T cells, surrounded by CXCL13 expressing cells. No accessory expression of BCL6 in lymphatic cells. Germinal centers are identifiable in routine light microscopy (see Pathology View, bottom left). An example for a BCL6^+^ secondary follicular TLS is given in **Figure 5A**. Images for each of the individual markers and their composites (for clarity without DAPI staining) are shown, together with the corresponding Pathology View (respective bottom left). Scale bars represent 100 µm. **(D)** Spatial annotation of tumor areas. Left: Definition of the invasive tumor front (red, 2) and the respective intra- and extratumoral compartments (brown, 1 and yellow, 3, respectively) in a tissue section of a human melanoma lymph node metastasis. Extratumoral compartments include site-specific and adipose tissue (yellow). Right: Allocation of intra- (brown/black) and extratumoral perimeters (green) of increasing radiuses (1 – max. 6 mm) drawn within and around the invasive tumor front. Counts and area of TLS were referred to the tissue area (yellow) within the respective intra- and extratumoral perimeters. TLS, tertiary lymphoid structures.

There are some reports on the presence of distinct TLS-associated cell types in primary human melanoma, but no systematic analysis of TLS phenotypes. Another difficulty is a lack of consensus on how to determine tumor-associated TLS density and distribution by a standardized evaluation methodology (33). To overcome the limitations of light microscopy, we performed 7-color multiplex immunohistochemistry on whole tissue sections from 103 human cutaneous melanoma samples with antibodies against TLS-defining molecular and cellular components: CXCL13, a key chemoattractant orchestrating the cellular composition of TLS, CD20 and CD4 for detection of B cells and T(helper) cells, CD21 and CD23 for the presence of a network of immature and mature follicular dendritic cells (FDCs), respectively, and BCL6, a key transcriptional regulator in B cells and T cells for germinal center formation. Representative stainings for detection of early, primary and secondary follicular TLS are given in **Figures 1A–C**.

We further specified the scoring of TLS density, relative area and spatial distribution by normalizing TLS counts and area to the tissue area (mm^2^) within defined extra- and intratumoral perimeters around the invasive tumor front (**Figure 1D**). Routine FFPE sections allowed the application of perimeters to the extratumoral compartment ranging from 1 to 6 mm with a median 6 mm distance in primary melanomas and 5 mm distance in metastatic melanomas. In the intratumoral compartment, we detected most TLS within the 1 and 2 mm perimeters; further evaluation within perimeters of 3 and more mm proved difficult because of small tumor size or presence of regions of necrosis, ulceration and hemorrhage which were excluded from the analysis. We, therefore, subdivided the intratumoral compartment into 1 and >1 mm perimeter areas (**Supplementary Figure 1**).

This allowed us *(i)* to analyze TLS phenotypes, density and spatial distribution in primary human melanoma and the association with established prognostic clinicopathologic factors; *(ii)* to compare TLS in terms of phenotype, density and spatial distribution in different stages of disease progression; *(iii)* to describe TLS for phenotype, density and spatial distribution in metastases from different body sites; and *(iv)* to characterize the cellular composition and spatial distribution of TLS-defining cell types in human melanoma compared to secondary lymphoid organs.

### Primary Human Melanomas Contain Mostly TLS of an Early Immature Phenotype

Here we demonstrate an evaluation strategy based on referring TLS counts and area to the tissue area (mm^2^) to determine TLS density and relative area, respectively, within defined intra- and extratumoral perimeters around the invasive tumor front (**Figure 1D**). This strategy should allow standardized read-out with high reproducibility.

**Figure 2:**
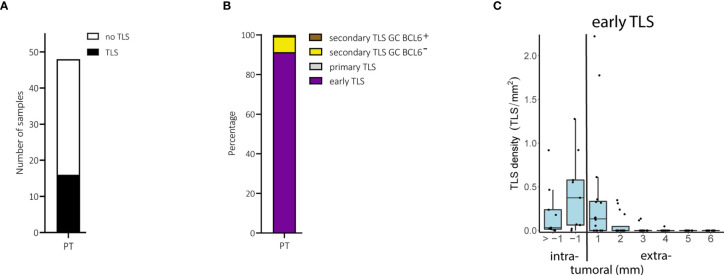
Primary melanomas (PT): TLS phenotypes, density and spatial distribution. **(A)** Prevalence of TLS in primary tumor samples. **(B)** Relative prevalence of TLS phenotypes in primary tumors. **(C)** Density of early TLS over intra- and extratumoral compartments of primary tumors. In all boxplots, lower and upper hinges correspond to the first and third quartiles, center line to the median. Upper and lower whisker extend from the hinge to the largest value no further than 1.5 times the interquartile range. Values outside this range are shown as outliers (black circles). Individual patient values are shown as black dots. TLS, tertiary lymphoid structures; GC, germinal center.

We found TLS in only 16 of 48 (33.3%) primary melanomas (**Figure 2A**). These TLS were predominantly of the early TLS phenotype (n=241 of a total 264; 91.3%; **Figure 2B**). Only four tumor samples presented with a secondary follicular TLS and one of these had BCL6^+^ lymphatic cells. Primary follicular TLS were not identified (**Figure 2B**).

The density of early TLS ranged from 0 to 3.0 per intratumoral mm^2^ and 0 to 1.1 per extratumoral mm^2^; their relative area ranged from 0 to 9.3% of the intratumoral compartment and 0 to 3.3% of the extratumoral compartment. Comparison of areas of 1 and >1 mm perimeters in the intratumoral compartment was possible only in primary melanomas with a Breslow depth of ≥2 mm and the presence of TLS (n=9). In these samples, early TLS appeared at a higher density within the 1 mm perimeter of the intratumoral compartment (**Figure 2C**), often located directly at the inner invasive tumor front and/or between tumor cells without a preferential confinement to stromal septa (**Supplementary Figure 2A**). The extratumoral compartment could be analyzed in all 16 primary tumors with TLS. Extratumoral TLS were mostly present within the 1 mm perimeter with a drop in density with the 2 to 6 mm perimeters (**Figure 2C**). Densities, relative areas and spatial distribution for TLS in primary melanomas are given in **Supplementary Table 1**.

When we stratified the primary melanoma samples for the prognostically most important parameter, *i.e.* metastasis, we found no differences for the presence of TLS (P = 0.75, Fisher’s exact test), or for their density, relative area and spatial distribution. The same was true for other prognostically important categorical clinicopathologic parameters such as Breslow depth, ulceration, age and sex.

Thus, only a subgroup of primary human melanoma contains TLS and if they do, they are mainly of an immature early phenotype. TLS mostly occur intratumorally within a distance of 1 mm to the invasive tumor front and their presence is not associated with prognostic clinicopathologic factors.

### Melanoma Disease Progression Is Associated With an Increased Density of Extratumoral Secondary Follicular TLS

TLS phenotypes and density can vary between cancer stages and body sites. We, therefore, compared primary human melanomas with melanoma metastases, including early locoregional and late distant metastases.

**Figure 3:**
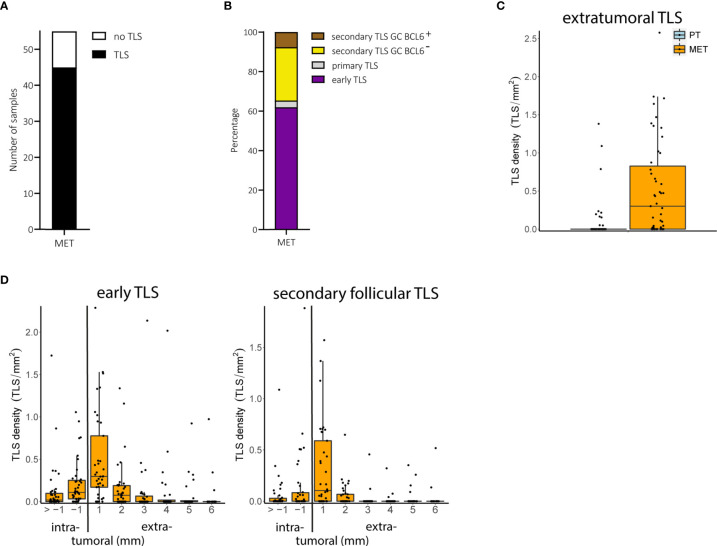
Melanoma metastases (MET): TLS phenotypes, density and spatial distribution. **(A)** Prevalence of TLS in metastatic tumor samples. **(B)** Relative prevalence of TLS phenotypes in melanoma metastases. **(C)** Comparison of extratumoral TLS density with primary melanomas. **(D)** Densities of early TLS (left) and secondary follicular TLS (right) over intra- and extratumoral compartments of metastatic tumors. In all boxplots, lower and upper hinges correspond to the first and third quartiles, center line to the median. Upper and lower whisker extend from the hinge to the largest value no further than 1.5 times the interquartile range. Values outside this range are shown as outliers (black circles). Individual patient values are shown as black dots. TLS, tertiary lymphoid structures; GC, germinal center.

We found TLS in 45 of 55 (81.2%) metastatic melanoma samples (**Figure 3A**). Early TLS were the most prevalent TLS phenotype, followed by secondary follicular TLS with a BCL6^-^ germinal center and only small proportions of both secondary follicular TLS with a BCL6^+^ germinal center and primary follicular TLS (**Figure 3B**). While secondary follicular TLS were rarely found in primary tumors, they were present in 54.5% of metastatic tumors. These secondary follicular TLS appeared in the tumor stroma, in stromal septa through the intratumoral compartment surrounding tumor nests, and in the peritumoral stroma at the invasive tumor front (**Supplementary Figure 2B**). TLS density in melanoma metastases ranged from 0 to 2.2, median 0.04 per intratumoral mm^2^ and 0 to 2.6, median 0.30 per extratumoral mm^2^; the relative area of TLS from 0 to 17.3%, median 0.06% of the intratumoral compartment and 0 to 16.8%, median 0.76% of the extratumoral compartment.

The number of tumor samples with TLS did not differ between early locoregional and late distant metastases (P = 0.45, Fisher’s exact test) but was significantly increased in metastatic tumors compared with primary tumors (P < 0.001, Fisher’s exact test). Both TLS density and relative area - particularly in the extratumoral compartment - were also significantly increased in metastatic samples compared to primary tumors (FDR < 0.001 for both, Wilcoxon rank sum test; **Figure 3C**), mainly driven by early and secondary follicular TLS (5.9 and 34.9 fold increase in counts for early and secondary follicular TLS, respectively). Both early and secondary follicular TLS were present throughout the entire intratumoral compartment, with a higher density within the 1 mm perimeter. Extratumoral TLS were mostly present within the 1 mm perimeter, with a drop in density with the 2 to 6 mm perimeters (**Figure 3D**). Densities, relative areas and spatial distribution for TLS in melanoma metastases are given in **Supplementary Table 1**.

Thus, metastatic melanoma disease differs from local disease in the presence particularly of secondary follicular TLS phenotypes, most of which are found within the extratumoral compartment of 1 mm distance to the invasive tumor front.

### Metastatic Sites Differ for Density and Spatial Distribution of Secondary Follicular TLS

When we further compared early locoregional and late distant metastatic tumors, we observed an increase in the density of particularly extratumoral secondary follicular TLS and BCL6^-^ germinal centers in late distant metastatic tumors (P = 0.05 and 0.03, FDR = 1.0 and 0.875, respectively, Wilcoxon’s rank sum test). Because early locoregional metastatic tumors were enriched for lymph node tissues, we hypothesized that a different representation of tumor sites could drive this effect.

**Figure 4:**
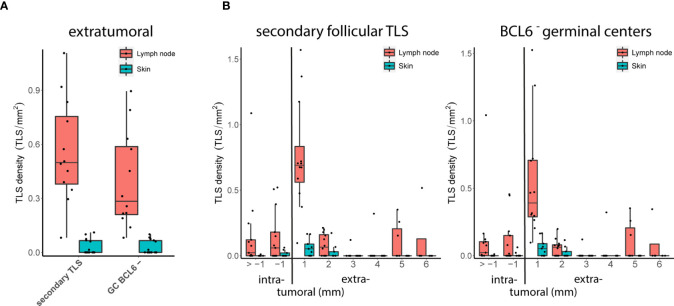
Metastatic tumor sites: TLS density and spatial distribution. **(A)** Densities of extratumoral secondary follicular TLS (left) and BCL6^-^ germinal centers (GC BCL6^-^, right) in distant metastatic lymph node and skin sites. **(B)** Densities of secondary follicular TLS (left) and BCL6^-^ germinal centers (right) over intra- and extratumoral compartments in distant metastatic lymph node and skin sites. In all boxplots, lower and upper hinges correspond to the first and third quartiles, center line to the median. Upper and lower whisker extend from the hinge to the largest value no further than 1.5 times the interquartile range. Values outside this range are shown as outliers (black circles). Individual patient values are shown as black dots.

Distant metastatic samples contained comparable numbers of lymph node and skin metastases. TLS could be detected in 13 of 13 (100%) distant metastatic lymph node samples compared with only 10 of 15 (66.7%) distant metastatic skin samples. Consistent with our hypothesis, we found an increased density of extratumoral secondary follicular TLS and extratumoral BCL6^-^ germinal centers in metastatic lymph node samples compared with metastatic skin samples (FDR = 0.06 both, Wilcoxon’s rank sum test; **Figure 4A**). We found no difference for extratumoral BCL6^+^ germinal centers, most likely due to their small sample size. At lymph node sites, the density of secondary follicular TLS and BCL6^-^ germinal centers was highest within the extratumoral 1 mm perimeter with a drop with the extratumoral 2 to 6 mm perimeters and the intratumoral compartment (**Figure 4B**). At skin sites, secondary follicular TLS and BCL6^-^ germinal centers were almost exclusively present within the 1 and 2 mm extratumoral perimeters (**Figure 4B**).

A similar comparison for samples from the cohort with early locoregional metastases was not possible because of the lack of a sufficient number of skin metastases. However, the density of extratumoral secondary follicular TLS and extratumoral BCL6^-^ germinal centers in early locoregional lymph node samples was lower than in distant late lymph node metastases (FDR = 0.002 and 0.003, respectively, Wilcoxon’s rank sum test) and was not different to distant late skin metastases (FDR = 0.29 both, Wilcoxon’s rank sum test). In the small number of melanoma brain metastases (n=3), secondary follicular TLS - and thus BCL6^-^ germinal centers - were completely absent. TLS phenotypes, densities and spatial distribution for metastatic tumor samples are given in **Supplementary Table 1**.

Thus, depending on the tumor site, human melanoma metastases vary in terms of TLS density, particularly of extratumoral secondary follicular TLS with BCL6^-^ germinal centers, and spatial distribution within intra- and extratumoral compartments.

### Secondary Follicular TLS From Human Melanoma Mostly Lack Germinal Center Polarity

**Figure 5:**
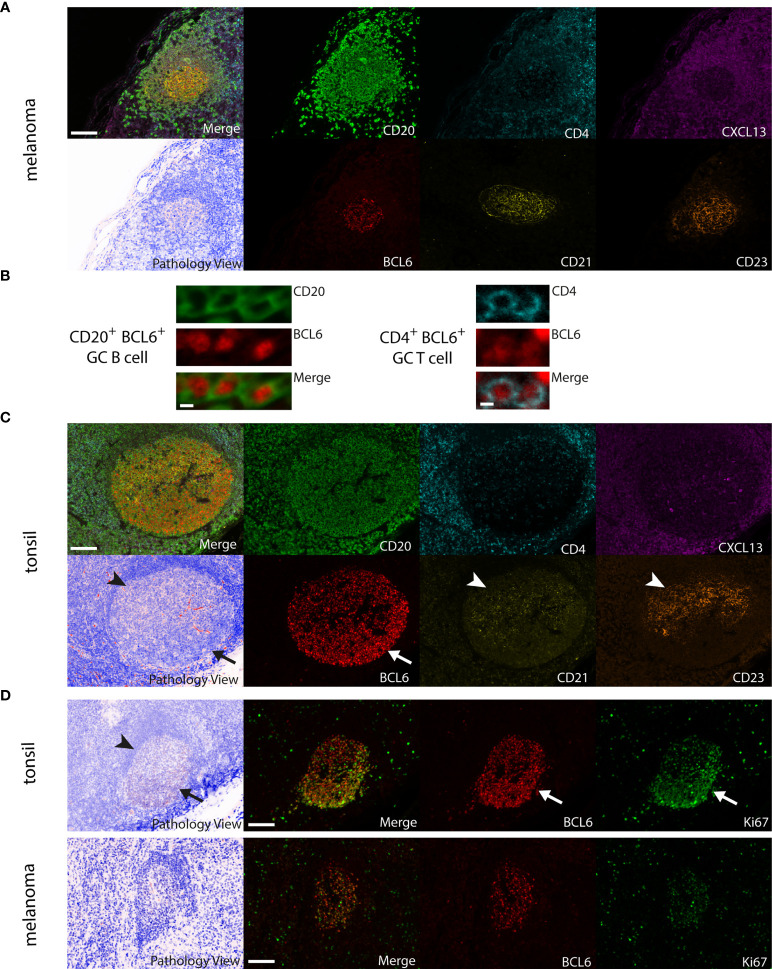
Comparison of large BCL6^+^ secondary follicular TLS in human melanoma to tonsil tissue. **(A)** Melanoma: CD20^+^ lymphocyte aggregates with a germinal center surrounded by CXCL13 expressing cells. A CD21^+^ and CD23^+^ FDC network covers the complete germinal center area with randomly interspersed CD4^+^ T cells and BCL6^+^ cells. No detectable germinal center polarity, no identifiable light and dark zone areas in routine light microscopy (see Pathology View, bottom left). **(B)** Close up of BCL6^+^CD20^+^ B cells and BCL6^+^CD4^+^ T cells from a BCL6^+^ secondary follicular TLS in human melanoma. **(C)** Tonsil: note canonical germinal center polarity with polarized spatial distribution of the CD21^+^ and CD23^+^ FDC network together with CD4^+^ T cells (arrowheads) and a complementary localization of BCL6^+^ cells (arrow). Identifiable light (arrowhead) and dark zone (arrow) areas in routine light microscopy (see Pathology View, bottom left). **(D)** Tonsil (upper row): polarized distribution of BCL6^+^, Ki67^+^ (arrows) and BCL6^+^Ki67^+^ cells in germinal centers with canonical germinal center polarity and the presence of light (arrowhead) and dark zone (arrow) areas in routine light microscopy (see Pathology View, left). Melanoma (lower row): note decreased numbers and absence of polarized distribution of BCL6^+^, Ki67^+^ and BCL6^+^Ki67^+^ cells in melanoma germinal centers. Lack of canonical germinal center polarity and absence of light and dark zone areas in routine light microscopy (see Pathology View, left). Images for each of the individual markers and their composites (for clarity without DAPI staining) are shown, together with the corresponding Pathology View (respective (bottom) left). Scale bars represent 100 µm **(A, C, D)** and 5 µm **(B)**.

Secondary follicular TLS in human melanoma metastases regularly contained key cellular and molecular components in a typical spatial arrangement, but only some larger secondary follicular TLS (21.7%) contained germinal centers that included BCL6^+^CD20^+^ B cells with interspersed BCL6^+^CD4^+^ T cells (**Figures 5A, B**). Interestingly, both BCL6^+^CD20^+^ B cells and BCL6^+^CD4^+^ T cells were located within the CD21^+^ and CD23^+^ FDC network, which covered the entire germinal center area (**Figure 5A**). This distinct spatial arrangement differed markedly from that in canonical germinal centers of secondary lymphoid organs such as tonsillar tissue. Here, BCL6^+^ secondary follicular structures regularly contained polarized germinal centers with the presence of dark and light zone areas. These were recognizable by light microscopy (**Figure 5C**, Pathology View) and characterized by a polarized distribution of the CD21^+^ and CD23^+^ FDC network together with CD4^+^ T cells and a discernable complementary distribution of BCL6^+^ lymphatic cells (**Figure 5C**). Tonsil germinal centers were further enriched for BCL6^+^Ki67^+^ cells with polarized distribution (**Figure 5D**, upper row). In contrast, melanoma germinal centers were smaller and had lower numbers of BCL6^+^, Ki67^+^ and BCL6^+^Ki67^+^ cells without polarized distribution (**Figure 5D**, lower row). Interestingly, BCL6^-^ melanoma germinal centers showed no enrichment for Ki67^+^ cells.

Thus, TLS in melanoma metastases reconcile the expression and spatial distribution of TLS-defining molecular and cellular markers in many aspects. However, they often lack germinal center polarity with the formation of proliferative dark zone areas enriched for BCL6^+^Ki67^+^ cells.

## Discussion

Here we show, *(i)* that only a subgroup of primary human melanomas contains TLS. These TLS mostly appear intratumorally within a 1 mm distance to the invasive tumor front, rarely form secondary lymphoid structures, and their presence seems not to be associated with prognostic factors; *(ii)* that melanoma metastases exhibit an increased TLS density, particularly of early and secondary follicular TLS, which are mostly found within an extratumoral 1 mm distance; *(iii)* that the density of secondary follicular TLS in metastases varies with time and disease site; and *(iv)* that secondary follicular TLS in melanoma often lack the presence of BCL6^+^ lymphatic cells as well as germinal center polarity with the formation of dark and light zone areas.

Studies on TLS in human cancer suffer from the lack of general agreement on the definition of TLS and their quantification. This may explain why, besides reports on a positive association of the presence or density of TLS with a favorable disease outcome in several human cancers, there are also reported negative associations. This discrepancy has been linked to differences in phenotype and spatial distribution of TLS as well as disease stages (19, 33, 34). Previous studies quantified TLS density in various ways, including counting TLS within intra- and/or extratumoral tumor areas, either directly at the invasive tumor front or within different distances from it. These counts were related to the length of the invasive tumor front and to a so-called immunoreactive area around the invasive front, or were not allocated to any quantified tissue area at all [reviewed in (9, 10, 27, 28, 33, 35)]. While these differences limit the reproducibility and comparability of the data, one important advantage of our study is the use of a standardized evaluation strategy based on referring TLS counts to tissue area (mm^2^) within defined intra- and extratumoral perimeters around the invasive tumor front. Another important asset is the use of multiplex immunohistochemistry for the characterization of TLS phenotypes. With the successive identification of molecular and cellular components defining and shaping TLS functionality (34) and the ability to simultaneously detect complex TLS marker combinations in histological sections (27, 28, 33), important progress has been made in identification and phenotyping of TLS in human tumor tissues. Here we applied multiplex immunohistochemistry for the simultaneous detection of six established TLS-defining molecular and cellular components (27, 28, 33, 34) and used at least two of these molecular and/or cellular markers in combination with structural features to characterize the individual TLS phenotypes. The advantage of this strategy is best exemplified by the identification of large secondary follicular TLS with germinal centers lacking the opposing polarization of CD21^+^ and CD23^+^ FDC networks along with CD4^+^ cells versus BCL6^+^ lymphatic cells with high proliferative activity as indicated by additional Ki67 staining. Given the prospectively high reproducibility of this standardized quantitative assessment approach for multiple marker expression in tumor tissues, our study may contribute to the development of a future widely accepted standard procedure for the analysis of TLS phenotypes, density and spatial distribution in human cancer.

Among various other cell types, the presence of high endothelial venules, mature dendritic cells and B cell aggregates can be indicative for the development or presence of TLS. In a small number of studies in primary cutaneous melanoma, MECA-79^+^ high endothelial venules have been described to co-localize with mature DC-LAMP^+^ DCs and dense lymphocyte infiltrates with CD20^+^ B cells, and their numbers were associated with tumor regression and low Breslow depth (23). Both MECA-79^+^ high endothelial venules and mature DC-LAMP^+^ DCs were detected at the invasive tumor front exclusively within the extratumoral area, an observation earlier reported for mature DC-LAMP^+^ DCs and associated dense lymphocyte infiltrates (17). In a later study by the same group, some dense lymphocyte infiltrates were characterized as follicular B cell aggregates, but their presence was not associated with improved survival (4). It is difficult to distinguish localized immune cell infiltrates from TLS and it has been subsequently shown that the presence of MECA-79^+^ high endothelial venules and mature DC-LAMP^+^ DCs is not sufficient to serve as surrogate parameter for the presence of TLS (12). In line with the latter observation, we found little primary or secondary follicular TLS in primary melanoma. However, in agreement with all these reports, we found dense CD20^+^ B cell aggregates together with CXCL13-secreting cells and some interspersed CD4^+^ T cells, which we termed early TLS.

Tumor-associated TLS are generally found in the extratumoral area (19, 36), although there are some exceptions (19, 36, 37). Interestingly, human melanoma metastases and a rare histotype of primary melanoma, namely desmoplastic melanoma, have recently been reported to contain intra- and extratumoral TLS (33, 38). We can now complement these reports with our observation on the presence of early TLS in intra- and extratumoral areas adjacent to the invasive tumor front in other histotypes. Neither density, nor area and distribution of early TLS were associated with metastasis or with other established prognostic parameters, an observation consistent with recent reports highlighting the prognostic utility particularly of mature TLS for disease and therapy outcome (9–11, 19, 27, 28).

In our study, metastases differed from local disease particularly in the presence of mature secondary follicular TLS phenotypes with germinal centers near the invasive tumor front. The presence of such secondary follicular TLS may have significant implications. In secondary lymphoid organs, such mature lymphoid structures are the site of clonal expansion, somatic hypermutation and affinity maturation of B cells and their differentiation into class-switched memory B cells and antibody secreting cells. In all likelihood, this is also true for TLS in human melanomas, as indicated by clonal amplification, somatic mutation and isotype switching of B cells in microdissected TLS from skin metastases (12) and significantly increased clonal counts for both heavy and light immunoglobulin chains and increased B cell receptor diversity in lymph node metastases with an increased density of secondary follicular TLS (10). The presence of TLS in human melanoma samples further correlates with increased frequencies of switched memory B cells, plasmablasts/plasma cells and less exhausted, activated memory-like CD4^+^ and CD8^+^ T cells (7, 9, 10) as well as with response to ICB therapy (9, 10).

Our data also indicate that the presence of secondary follicular TLS varies with time and site of metastasis. Originally it was reported that fully developed TLS are only present in melanoma metastases from skin, but not at several other sites (12). More recent reports, however, complemented this data for the presence of mature TLS at metastatic lymph node sites, but differed in the presence at extra-nodal sites (9, 10). In our study, the density of extratumoral secondary follicular TLS increased significantly from early regional to late distant lymph node metastasis. Distant metastases at lymph node sites also contained secondary follicular TLS at a higher density and in a different local distribution compared with skin sites. Whether these differences in TLS density and spatial distribution may reflect different immunological properties and perhaps a differential predictive value for patient survival and therapy response needs to be evaluated by site-to-site comparisons in future clinical studies. This is also true for assessing the predictive value of extratumoral TLS density, particularly within 1 mm distance to the invasive front of melanoma metastases. We further detected secondary follicular TLS in a small number of metastases from lung and kidney, but not from brain, bone and urinary bladder. These data are in line with previous reports on the absence of fully developed TLS in melanoma brain metastases (12, 39), but need to be reconciled in larger studies.

Lymph node, skin and lung metastases often exhibited TLS of different phenotypes, and a small but non-negligible number exhibited a BCL6^+^ germinal center. In line with the previously reported expression of germinal center initiating and polarizing gene signatures in B cells of human melanoma metastases (9), these BCL6^+^ melanoma TLS reconciled the expression of molecular and cellular markers that define mature secondary follicular structures in secondary lymphoid organs. However, melanoma TLS with BCL6^+^ germinal centers often lacked germinal center polarization despite the expression of BCL6 in some germinal center B cells and T cells. Compared with human tonsil, germinal centers from human melanoma generally contained reduced numbers of both BCL6^+^CD20^+^ B cells and BCL6^+^CD4^+^ T cells.

Polarized cell distribution is a key feature of a functional mature germinal center and BCL6 is an important regulator of germinal center initiation and maintenance through direct and indirect regulation of genes controlling proliferation, DNA damage response, apoptosis and cell differentiation (40). The generally low expression of BCL6 in our study argues against a highly proliferative activity of germinal center B cells. This assumption is further supported by our data on the rather small size of germinal centers in melanoma with frequent lack of germinal center polarization and low expression of Ki67 compared with secondary lymphoid organs. In canonical germinal centers from secondary lymphoid organs, particularly BCL6^+^ cells with polarized distribution are Ki67 positive. This may explain the recently published unexpectedly low number of Ki67^+^ cells with unpolarized distribution in melanoma germinal centers (9). In contrast, repression of BCL6 expression or activity in B cells is important for differentiation and exit from the germinal center. This BCL6 repression in germinal center B cells follows B cell receptor signaling through recognition of antigens presented by mature FDCs as well as T cell help through CD40 ligation. A fine-tuned balance between the strength of B cell receptor signaling and of T cell help ultimately determines the level of BCL6 expression and thus the fate of B cells, namely induction of apoptosis or differentiation into antibody secreting cells (34). Both lead to reduced BCL6^+^ B cell numbers as observed in our study. The expected continuous availability of tumor-associated antigens at melanoma sites together with our observation on the frequent lack of polarization of the mature FDC network rather argues more for a B cell receptor signaling-induced repression of BCL6 by comprehensive antigen presentation. Additional T cell help - as indicated by our observation on the colocalization with CD4^+^ T cells - may then activate NFkB/IRF4-promoted memory B cell and plasmablast/plasma cell differentiation rather than apoptosis. This view is further supported by the reported high frequencies of switched memory B cells, plasmablasts/plasma cells in human melanoma samples with mature TLS (9, 10). While these assumptions need further validation, they have some potentially important clinical implications for improving ICB therapy in melanoma, complementary to solely increasing TLS numbers. Given the already sizable numbers of early TLS and small secondary follicular TLS, our data highlight the importance of fostering TLS maturation to induce broad germinal centers with canonical germinal center polarity and the presence of light and dark zone areas for increased proliferation and differentiation of anti-tumor B cells. The rare observation of melanoma TLS with polarized germinal centers and Ki67^+^BCL6^+^ lymphatic cells gives a first hint that this ought to be possible.

## Conclusion

This study significantly widens the knowledge of TLS phenotypes, density and spatial distribution in human melanoma. Using multiplex immunohistochemistry for TLS-defining molecular and cellular components together with a standardized evaluation strategy, our data reveal disease progression- and site-dependent changes in TLS phenotypes, density and distribution, as well as an altered germinal center formation with the frequent absence of germinal center polarity. These observations provide important premises for the further development of cancer immunotherapy and suggest that - in addition to enhancing TLS density - strategies aimed at inducing TLS maturation should be considered. The presented assessment procedure may further contribute to the development of a future widely accepted evaluation standard for TLS analysis in human cancer.

## Data Availability Statement

The original contributions presented in the study are included in the article/**Supplementary Material**. Further inquiries can be directed to the corresponding author.

## Ethics Statement

The patients/participants provided their written informed consent to tumor sample collection. Tumor sample collection and the studies involving these tumor samples were reviewed and approved by the Ethikkommission Nordwest- und Zentralschweiz (votes BASEC 2016-01499 and 2019-00927). Tumor tissue analysis and read-out was additionally approved by the Ethics Committee of the Medical University of Vienna (ethics vote 1999/2019).

## Author Contributions

SW: conception. JG, FW, and SW: design. HL, KM, KG, FW, MS, and SW: clinical data collection and assembly, patient materials. KG and KM: Histopathology. FW, CW, MS, and SW: Multiplex immunostaining, automated imaging acquisition and data read-out. JG: Statistics. All authors contributed to the article and approved the submitted version.

## Funding

This work received support from the Austrian Science Fund (FWF) project P31127-B28 and IPPTO project number DOC 59-B33 to SW.

## Conflict of Interest

HL received travel grants and consultant fees from BMS and MSD. HL received research support from BMS, Anaveon, Glycoera and Palleon Pharmaceuticals.

The remaining authors declare that the research was conducted in the absence of any commercial or financial relationships that could be construed as a potential conflict of interest.
