# Supplementary material for: A Standardized Analysis of Tertiary Lymphoid Structures in Human Melanoma: Disease Progression- and Tumor Site-Associated Changes With Germinal Center Alteration
Source: Front Immunol. 2021 Jun 24;12:675146. doi: 10.3389/fimmu.2021.675146 (PMC8264652; doi:10.3389/fimmu.2021.675146)
Supplement: Supplementary file 1 [file DataSheet_1.docx]

Supplementary Material


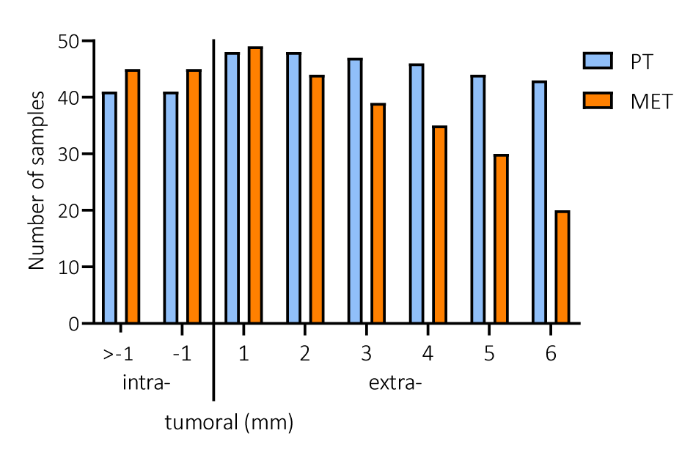


**Supplementary Figure 1.** Coverage of intra- and extratumoral compartments by the primary (PT) and metastatic (MET) tumor samples of our study.


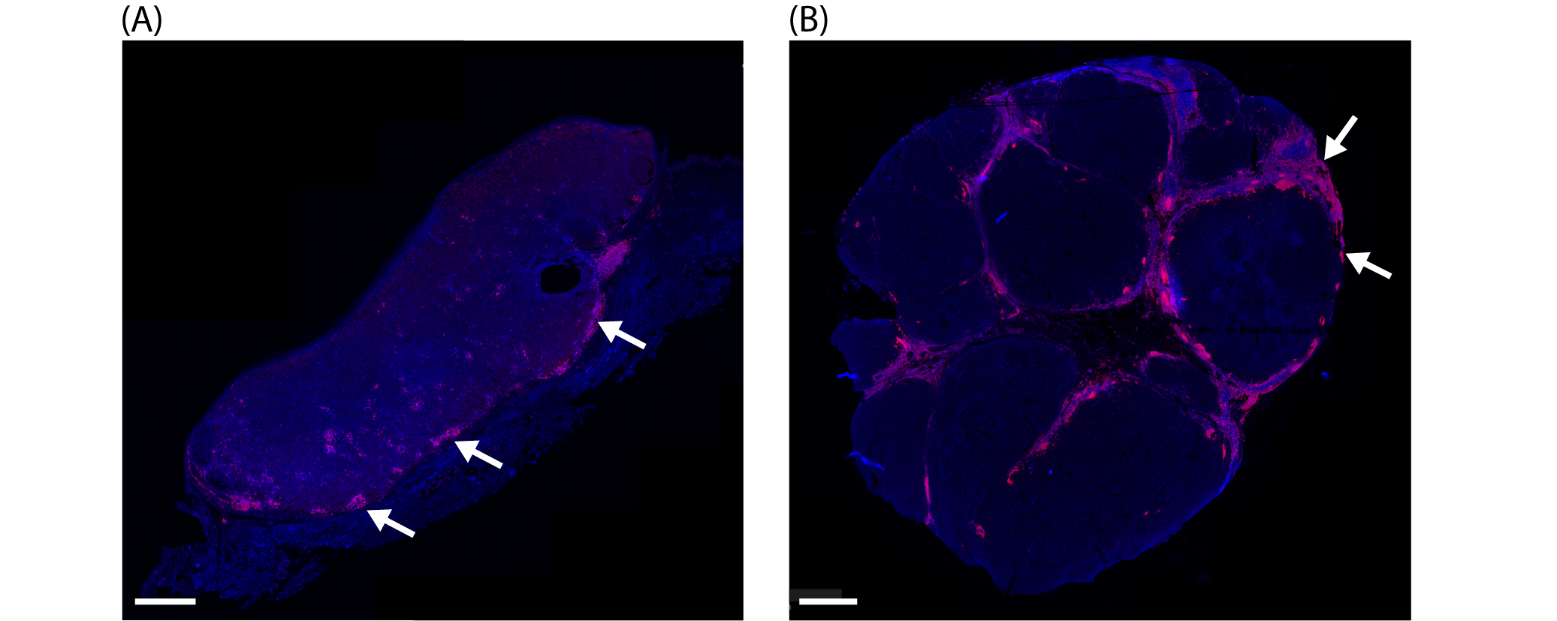


**Supplementary Figure 2.** Distribution of TLS in primary (PT) and metastatic (MET) tumor samples. (**A**) PT: intratumoral distribution of early TLS, note lymphocyte aggregates at the inner invasive tumor front (arrows) and between tumor cells without preferential confinement to stromal septa. Scale bar represents 1 mm. (**B**) MET: preferential distribution of large lymphocyte aggregates in stromal septa of the intratumoral compartment, often around tumor nests, as well as in the extratumoral stroma surrounding the invasive tumor front (arrows). Scale bar represents 2 mm. Immunofluorescence staining for CD20 (red) and DAPI staining (blue) from multiplex immunohistochemistry are shown.

**Supplementary Table 1.** Compilation of all data for TLS phenotypes, densities and spatial distribution in primary and metastatic melanoma samples. (**A**) Counts and areas per TLS maturation stage with dedicated spatial distribution, patient IDs and disease stage. (**B**) Areas of intra- and extratumoral perimeters with dedicated patient IDs and disease stage. (“no entry” = not present, n.d. = not determined, PT non met = primary tumor without subsequent metastasis, PT met = primary tumor that metastasized, MET = metastasis).
